# Role of the Hepatorenal Index in the Detection of HbA1c-Defined Dysglycemia in Patients With Non-alcoholic Fatty Liver Disease: A Cross-Sectional Study

**DOI:** 10.7759/cureus.116102

**Published:** 2026-09-11

**Authors:** Geethu Divakar, Sarin Vidya, Afrah Fathima Karimbanakkal Edakkattu, Nisar Karekadavath

**Affiliations:** 1 Department of Radiodiagnosis, Government Medical College Manjeri, Manjeri, IND; 2 Department of Radiodiagnosis, Government Medical College Kozhikode, Kozhikode, IND; 3 Department of Medicine, Government Medical College Manjeri, Manjeri, IND

**Keywords:** dysglycemia, hba1c, hepatic steatosis, hepatorenal index, metabolic dysfunction-associated steatotic liver disease, non-alcoholic fatty liver disease, ultrasonography

## Abstract

Background

Non-alcoholic fatty liver disease (NAFLD), increasingly discussed within the metabolic dysfunction-associated steatotic liver disease (MASLD) framework, is closely linked to insulin resistance and abnormal glucose metabolism. The hepatorenal index (HRI) provides a quantitative ultrasound estimate of hepatic steatosis, but its value for detecting HbA1c-defined dysglycemia remains uncertain.

Objective

The primary objective was to estimate the prevalence of HbA1c-defined dysglycemia among adults with ultrasonographically diagnosed NAFLD. Secondary objectives were to assess the association between HRI and HbA1c and to evaluate the independent association and discriminatory performance of HRI for dysglycemia.

Methods

This cross-sectional study included 116 adults with NAFLD evaluated at Government Medical College Manjeri, India, from August 2025 to February 2026. HRI was calculated from standardized B-mode ultrasound images using liver-to-right renal cortical echogenicity ratios, and the mean of three paired measurements was used for analysis. Dysglycemia was defined as HbA1c ≥5.7%, comprising prediabetes-range HbA1c (5.7-6.4%) and diabetes-range HbA1c (≥6.5%). Pearson and Spearman correlations, linear and robust regression, logistic regression, and receiver operating characteristic (ROC) analyses were performed.

Results

HbA1c-defined dysglycemia was present in 69 of 116 participants (59.5%), including 36 (31.0%) with prediabetes-range HbA1c and 33 (28.4%) with diabetes-range HbA1c. Mean HRI was 1.54 (SD 0.28). HRI showed a weak positive Pearson correlation with HbA1c (r = 0.232; p = 0.012), whereas Spearman correlation was not significant (rho = 0.111; p = 0.237). The association was attenuated in Huber robust regression and was not statistically significant after adjustment for age, sex, and body mass index (BMI) (beta = 0.41; 95% CI -0.19 to 1.01; p = 0.180). HRI was not independently associated with HbA1c-defined dysglycemia in adjusted logistic regression (OR per 0.1-unit increase = 1.05; 95% CI 0.91 to 1.21; p = 0.501). ROC analysis showed poor standalone discrimination (AUC = 0.547; 95% CI 0.433 to 0.653; p = 0.393). The sample-derived Youden threshold of HRI ≥1.71 had a sensitivity of 40.6% and specificity of 78.7% and was considered exploratory.

Conclusion

HRI showed at most a weak, method-sensitive association with HbA1c and poor standalone discrimination for HbA1c-defined dysglycemia. HRI may support sonographic quantification of hepatic steatosis but should not replace direct biochemical screening for dysglycemia.

## Introduction

Non-alcoholic fatty liver disease (NAFLD) is one of the most common chronic liver conditions worldwide and is increasingly recognized as part of a systemic metabolic disease spectrum. Contemporary nomenclature has shifted toward metabolic dysfunction-associated steatotic liver disease (MASLD), emphasizing the central role of cardiometabolic risk rather than defining the disease only by exclusion of alcohol-related liver injury [1,2]. Although current nomenclature favours MASLD, the term NAFLD is retained in this study because participant identification and eligibility were based on the prespecified NAFLD definition used in the study protocol. The complete MASLD diagnostic framework was not prospectively applied; therefore, retrospective reclassification could introduce misclassification.

NAFLD is closely linked to insulin resistance, visceral adiposity, dyslipidemia, hypertension, and type 2 diabetes mellitus. Hepatic fat accumulation can worsen hepatic insulin sensitivity and contribute to systemic glucose dysregulation, while chronic dysglycemia can accelerate progression from simple steatosis toward steatohepatitis, fibrosis, and adverse cardiovascular outcomes [3,4]. This bidirectional relationship makes metabolic screening clinically important when hepatic steatosis is identified.

Current clinical evaluation of patients with steatotic liver disease includes assessment of cardiometabolic risk factors together with liver enzymes, lipid profile, and glycemic status. Non-invasive fibrosis scores, particularly the fibrosis-4 index (FIB-4), are commonly used for initial risk stratification for advanced fibrosis, with elastography or specialist assessment considered when indicated [2]. These assessments address different components of the disease: biochemical tests characterize metabolic and hepatic abnormalities, whereas FIB-4 estimates fibrosis risk. None directly quantifies hepatic fat, supporting the continued clinical relevance of accessible imaging-based assessment.

Ultrasonography remains the most accessible and practical first-line imaging modality for fatty liver assessment in routine clinical settings. Conventional visual grading is inexpensive and widely available but remains semi-quantitative and operator-dependent. The hepatorenal index (HRI), calculated as the ratio of liver parenchymal echogenicity to right renal cortical echogenicity, was developed to provide a more objective sonographic estimate of hepatic steatosis [5-8].

Previous studies have supported HRI as a useful marker for detecting and grading hepatic steatosis. Some evidence has also suggested a relationship between HRI and impaired glucose metabolism in NAFLD patients [9]. However, hepatic echogenicity and systemic glycemic status are related but not interchangeable clinical outcomes. A marker that quantifies steatosis may correlate weakly with HbA1c without providing clinically acceptable discrimination for dysglycemia.

Although HRI is useful for quantifying hepatic steatosis, its ability to identify dysglycemia among patients with NAFLD remains uncertain, particularly in real-world, hospital-based populations. The primary objective of this study was to estimate the prevalence of HbA1c-defined dysglycemia among adults with ultrasonographically diagnosed NAFLD. The secondary objectives were to assess the association between ultrasound-derived HRI and HbA1c levels and to evaluate the independent association and discriminatory performance of HRI for HbA1c-defined dysglycemia.

## Materials and methods

Study design, setting, and participants

This observational cross-sectional study was conducted in the Department of Radiodiagnosis, Government Medical College Manjeri, Kerala, India, from August 2025 to February 2026. Consecutive eligible adult patients with ultrasonography-diagnosed NAFLD were enrolled after institutional approval and informed consent.

Inclusion criteria were age 18 years or above, ultrasound-based diagnosis of NAFLD, recorded subcutaneous tissue thickness according to protocol requirements, and willingness to provide written informed consent. Exclusion criteria were significant alcohol consumption (>20 g/day for females and >30 g/day for males), other known causes of chronic liver disease, previously diagnosed diabetes mellitus or current antidiabetic medication use, severe heart disease, kidney failure, pregnancy, abnormal renal cortical echogenicity, or inadequate visualization of the right kidney. Because known diabetes and antidiabetic medication use were exclusion criteria, HbA1c values ≥ 6.5% in the analytical dataset were interpreted as diabetes-range glycemia rather than established diabetes unless confirmed separately by clinical evaluation or repeat diagnostic testing.

Sample size

The approved protocol calculated a minimum sample size of approximately 103 using the single-proportion formula [10]. The calculation assumed an expected prevalence of 48.31%, a 95% confidence level, and a relative precision of 20% of the expected prevalence, corresponding to an absolute margin of error of approximately 9.66% [9,10]. The sample size was calculated primarily to estimate the prevalence of impaired glycemic status; therefore, the association and receiver operating characteristic (ROC) analyses were considered exploratory.

Ethical approval and consent

Ethics Committee approval was granted by the Institutional Ethics Committee, Government Medical College Manjeri, Kerala, India (IEC/GMCM/178/2025; dated 16/06/2025). Written informed consent was obtained from all participants before enrollment. Patient confidentiality was maintained, and de-identified data were used for analysis.

Clinical and biochemical assessment

Demographic and clinical variables included age, sex, height, weight, body mass index (BMI), hypertension status, smoking status, and subcutaneous tissue thickness. Biochemical variables included HbA1c, alanine aminotransferase (ALT), aspartate aminotransferase (AST), total cholesterol, and triglycerides. HbA1c was measured by the institutional clinical laboratory using its routine standardized assay. Completeness of the variables used in the principal analyses was assessed before statistical analysis.

Ultrasound protocol and HRI calculation

All participants underwent fasting abdominal ultrasonography. B-mode ultrasound was performed using a Voluson E8 ultrasound system (GE Healthcare, Milwaukee, WI, USA) with a convex-array 2-5 MHz multifrequency probe. Gain, depth, focus, and time-gain compensation settings were standardized and maintained consistently during HRI image acquisition to minimize machine-setting-related variability (Figure 1).

**Figure 1:**
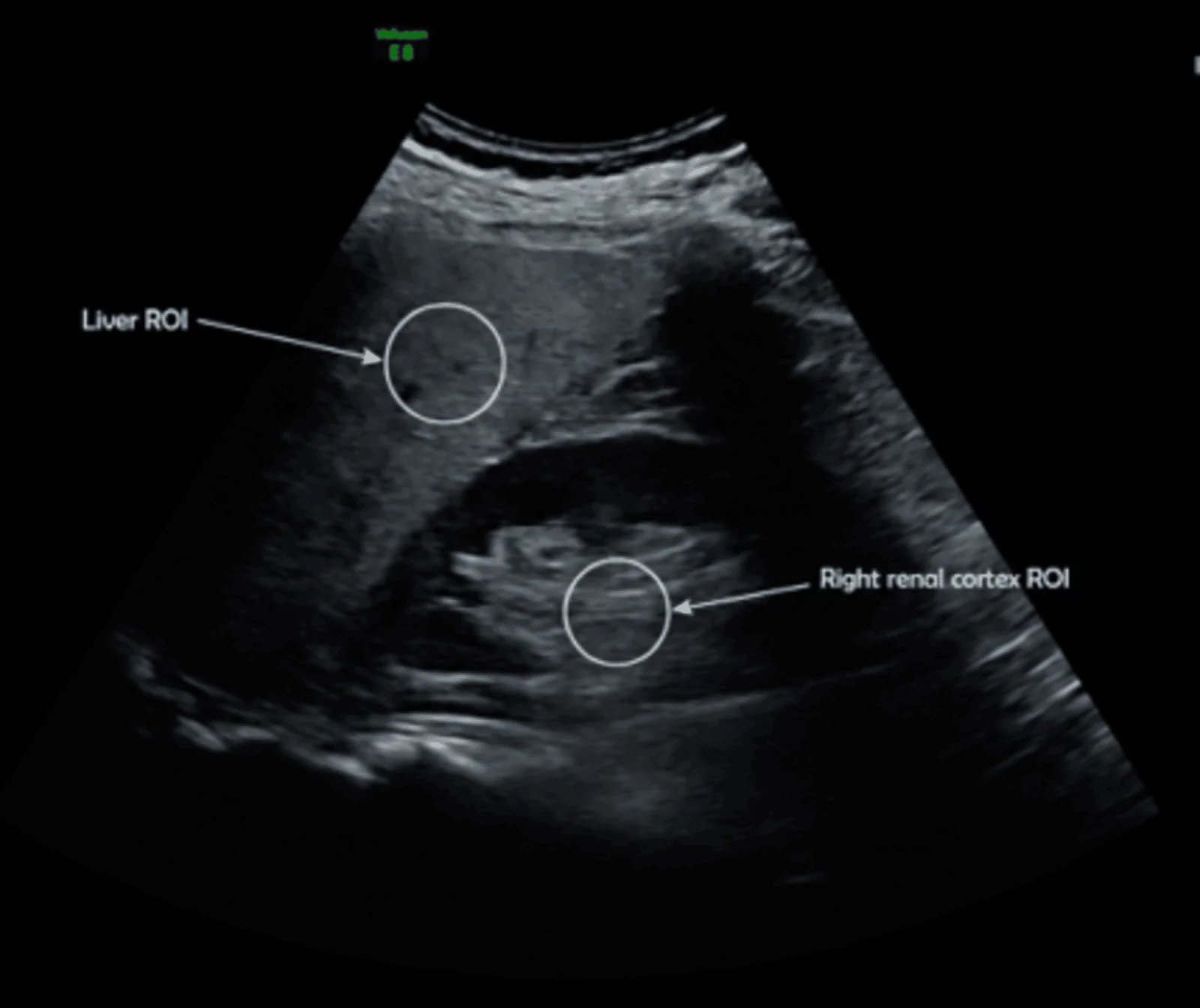
Representative B-mode ultrasound image showing region-of-interest placement for hepatorenal index measurement.

Stored images were analyzed using Fiji (ImageJ version 1.54r; National Institutes of Health, Bethesda, MD, USA). Fixed square regions of interest (ROIs) measuring 400 square pixels (20 × 20 pixels) were used for both hepatic and renal measurements. The hepatic ROI was placed within homogeneous parenchyma of the right hepatic lobe, typically segment VI. The renal ROI was placed within the right renal cortex at the upper pole. Hepatic and renal ROIs were positioned at comparable depths whenever technically feasible. Blood vessels, bile ducts, collecting-system structures, renal medullary pyramids, the renal sinus, focal lesions, perinephric fat, acoustic shadows, and other visible artifacts were avoided. Identical ROI dimensions were maintained for each paired hepatic and renal measurement. Mean grayscale intensity was recorded for each ROI using a standard 8-bit scale ranging from 0 to 255.

HRI was calculated by dividing the mean grayscale intensity of the hepatic ROI by that of the paired right renal cortical ROI. Three paired measurements were obtained through repeated ROI placements on the same image, and their arithmetic mean was used as the participant-level average HRI. All measurements were performed by a single radiologist with eight years of experience who was blinded to HbA1c values and other biochemical results. Formal intra-observer and inter-observer reproducibility assessments were not performed.

The protocol-defined HRI categories were mild steatosis (1.05-1.24), moderate steatosis (1.25-1.64), and severe steatosis (≥1.65), based on a previously published classification [9]. These categories were used for descriptive stratification and should not be interpreted as universally validated thresholds across different ultrasound systems and populations.

Definition of HbA1c-defined dysglycemia

For this study, HbA1c-defined dysglycemia was defined as an HbA1c value ≥5.7%, consistent with the American Diabetes Association criteria [11]. This composite category included prediabetes-range HbA1c (5.7%-6.4%) and diabetes-range HbA1c (≥6.5%). An HbA1c value <5.7% was classified as normal. Because participants with previously diagnosed diabetes or current antidiabetic medication use were excluded, values ≥6.5% were described as diabetes-range HbA1c and not as confirmed diabetes.

Statistical analysis

Statistical analyses were performed using R version 4.6.0 (R Foundation for Statistical Computing, Vienna, Austria). Continuous variables were summarized as mean with standard deviation (SD) or median with interquartile range (IQR), according to their distribution, and categorical variables were summarized as frequency and percentage. Data completeness was assessed before analysis. Between-group comparisons used Welch's t-test or the Mann-Whitney U test for continuous variables and the chi-square test or Fisher's exact test for categorical variables, as appropriate. For continuous baseline variables, between-group effect estimates were reported as mean differences, calculated as the HbA1c-defined dysglycemia group minus the normal-HbA1c group, with 95% confidence intervals (CIs). For categorical baseline variables, odds ratios (ORs) with 95% CIs were reported as effect estimates.

Associations between average HRI and continuous biochemical and metabolic variables were initially assessed using Pearson correlation. Because the HbA1c distribution included high values and showed evidence of skewness, Spearman rank correlation was also performed as a sensitivity analysis. The relationship between HRI and HbA1c was further evaluated using ordinary least-squares (OLS) linear regression, both unadjusted and adjusted for age, sex, and BMI.

Regression assumptions and model robustness were specifically evaluated because of the observed discordance between Pearson and Spearman correlation estimates. Linearity was assessed by visual inspection of scatterplots and residual patterns and by testing the addition of a quadratic HRI term. Residual distribution was assessed using residual diagnostic plots and the Shapiro-Wilk test. Heteroscedasticity was evaluated using residual-versus-fitted plots and the Breusch-Pagan test. Potentially influential observations were assessed using Cook's distance, with values exceeding 4/n used as a screening threshold for influential observations. Observations identified as influential were retained unless there was evidence of data-entry or measurement error.

To evaluate whether conventional linear-regression inference was sensitive to heteroscedasticity or influential observations, additional sensitivity analyses were performed. OLS regression was repeated using heteroscedasticity-consistent HC3 standard errors. Huber M-estimation robust regression was also performed to reduce the influence of observations with large residuals. Results from these sensitivity analyses were interpreted together with the Pearson and Spearman correlation estimates rather than relying solely on statistical significance from the conventional OLS model.

Logistic regression was used to evaluate HbA1c-defined dysglycemia as a binary outcome. Average HRI was first evaluated in an unadjusted model and then in a primary multivariable model adjusted for age, sex, and BMI. These covariates were retained based on clinical causal reasoning because age, sex, and overall adiposity may influence both hepatic steatosis and glycemic status. Covariate selection was not based on univariable statistical significance. Variables such as triglycerides, hypertension, subcutaneous tissue thickness, smoking, ALT, and AST may represent correlated metabolic characteristics, downstream manifestations, or overlapping measures of adiposity and were therefore not automatically included in the primary model. Given the sample size and the potential for overadjustment and model instability, the primary multivariable model was intentionally parsimonious.

An expanded exploratory sensitivity model additionally included triglycerides, hypertension, subcutaneous tissue thickness, current smoking, ALT, and AST to assess whether adjustment for these measured clinical and metabolic factors materially altered the association between HRI and dysglycemia. Because of the number of predictors relative to the sample size and the low frequency of some characteristics, particularly current smoking, this expanded model was considered a sensitivity analysis rather than the primary explanatory model. HRI ORs were expressed per 0.1-unit increase because a 1.0-unit change in HRI represents a clinically large difference.

ROC analysis was used to assess the standalone discriminatory ability of HRI for HbA1c-defined dysglycemia. The area under the ROC curve (AUC) and its 95% CI were estimated using the DeLong method. A sample-derived HRI threshold was identified using the Youden index. Because this threshold was selected and evaluated in the same cohort, it was regarded as exploratory and hypothesis-generating rather than as a clinically validated cutoff. Sensitivity, specificity, positive predictive value, negative predictive value, and overall accuracy were reported with 95% CIs using Wilson intervals. Positive and negative likelihood ratios were also calculated with 95% CIs using the logarithmic method.

All statistical tests were two-sided, and a p-value <0.05 was considered statistically significant. Because several association and sensitivity analyses were exploratory, emphasis was placed on effect estimates, confidence intervals, consistency across analytical approaches, and clinical interpretation rather than statistical significance alone. Reporting followed the Strengthening the Reporting of Observational Studies in Epidemiology (STROBE) recommendations [12,13].

## Results

Study population and prevalence of HbA1c-defined dysglycemia

The final analytical cohort included 116 patients with ultrasonographically diagnosed NAFLD and complete HbA1c and HRI data. Based on HbA1c criteria, 47 patients (40.5%) had normal HbA1c (<5.7%), whereas 69 (59.5%) had HbA1c-defined dysglycemia (HbA1c ≥5.7%). Within the dysglycemia group, 36 patients (31.0% of the total cohort) had prediabetes-range HbA1c (5.7%-6.4%), and 33 (28.4%) had diabetes-range HbA1c (≥6.5%). No missing observations were present for the variables reported in Table 1. The participant workflow is shown in Figure 2.

**Table 1:** Baseline characteristics by HbA1c-defined glycemic status. Data are mean (SD) or n (%). For continuous variables, the effect estimate is the mean difference (MD; dysglycemia minus normal HbA1c) with a 95% confidence interval. For categorical variables, the effect estimate is the odds ratio (OR) for HbA1c-defined dysglycemia comparing the stated category with its reference category (male sex, no hypertension, and non-current smoking, respectively). Continuous-variable comparisons used Welch t-tests; categorical p-values were retained from the prespecified chi-square/Fisher exact framework. ALT, alanine aminotransferase; AST, aspartate aminotransferase; BMI, body mass index; CI, confidence interval; HRI, hepatorenal index

Variable	Normal HbA1c (n=47)	HbA1c-defined dysglycemia (n=69)	Missing, n	Effect estimate (95% CI)	p-value
Age, years	42.53 (9.36)	46.75 (11.46)	0	MD 4.22 (0.38 to 8.07)	0.032
Height, m	1.639 (0.067)	1.640 (0.077)	0	MD 0.000 (-0.026 to 0.027)	0.975
Weight, kg	71.77 (13.80)	75.33 (11.33)	0	MD 3.57 (-1.27 to 8.40)	0.146
BMI, kg/m²	26.61 (4.23)	27.99 (3.55)	0	MD 1.38 (-0.12 to 2.87)	0.070
Subcutaneous tissue thickness, mm	33.07 (5.93)	36.00 (6.31)	0	MD 2.93 (0.65 to 5.21)	0.012
Average HRI	1.51 (0.26)	1.56 (0.30)	0	MD 0.05 (-0.05 to 0.16)	0.316
HbA1c, %	5.30 (0.29)	7.21 (1.97)	0	MD 1.91 (1.43 to 2.39)	<0.001
ALT, U/L	37.47 (29.18)	37.55 (31.06)	0	MD 0.08 (-11.15 to 11.32)	0.988
AST, U/L	23.79 (10.34)	24.26 (12.97)	0	MD 0.47 (-3.83 to 4.78)	0.828
Total cholesterol, mg/dL	197.34 (40.15)	211.74 (39.91)	0	MD 14.40 (-0.63 to 29.43)	0.060
Triglycerides, mg/dL	188.55 (88.49)	230.38 (131.14)	0	MD 41.82 (1.43 to 82.22)	0.043
Female sex, n (%)	21 (44.7)	42 (60.9)	0	OR 1.93 (0.91 to 4.08)	0.126
Hypertension, n (%)	6 (12.8)	16 (23.2)	0	OR 2.06 (0.74 to 5.74)	0.244
Current smoking, n (%)	5 (10.6)	2 (2.9)	0	OR 0.25 (0.05 to 1.35)	0.186

**Figure 2:**
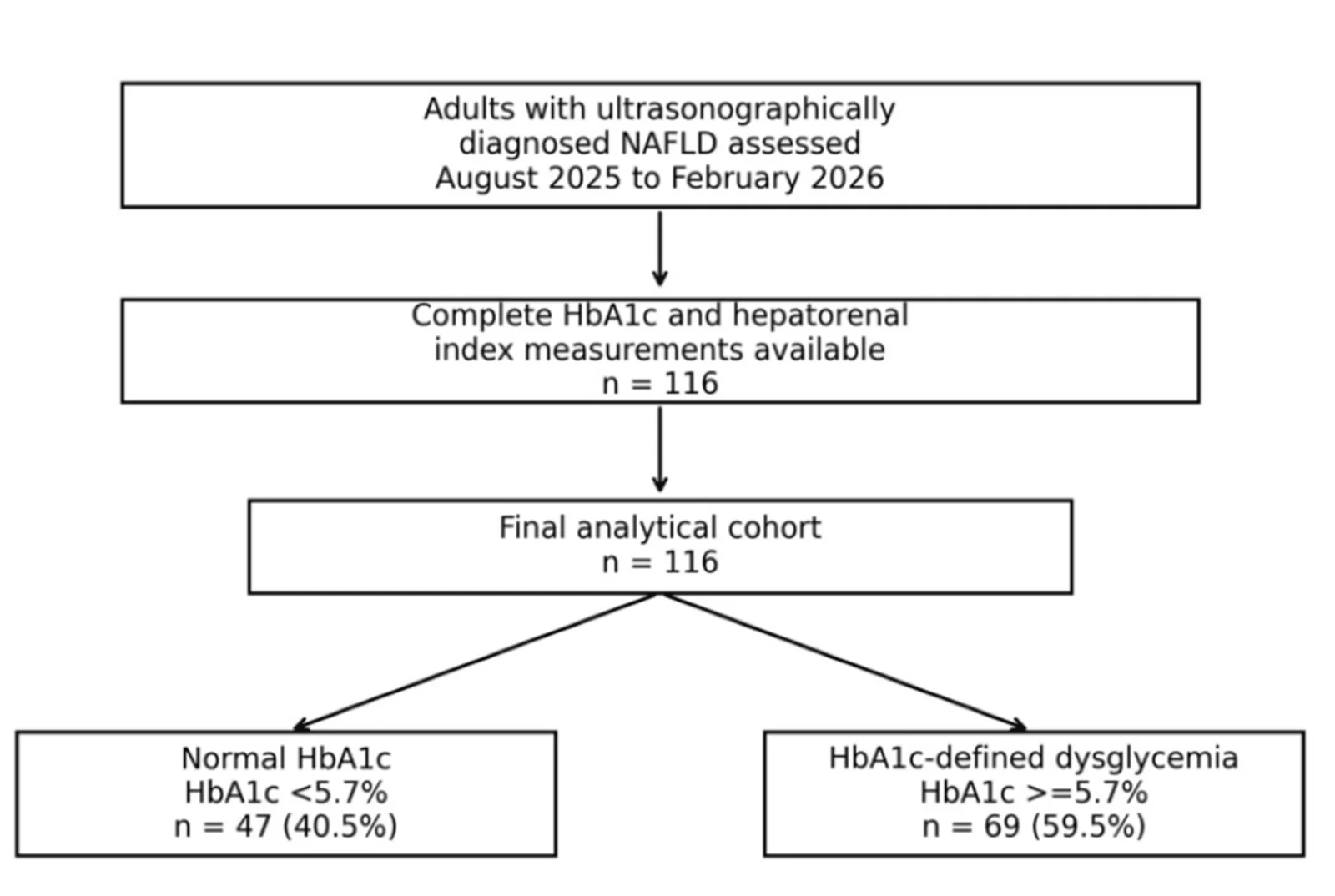
Participant inclusion and analytical classification workflow based on HbA1c-defined glycemic status. The final analytical cohort included 116 patients with complete HbA1c and hepatorenal index (HRI) measurements.

Baseline characteristics

Baseline demographic, anthropometric, biochemical, and ultrasonographic characteristics are presented in Table 1. Patients with HbA1c-defined dysglycemia were older than those with normal HbA1c (Welch's t(110.17) = 2.18, p = 0.032) and had greater subcutaneous tissue thickness (t(102.91) = 2.55, p = 0.012). Triglyceride levels were also higher in the dysglycemia group (t(114.00) = 2.05, p = 0.043). Differences in BMI (t(87.13) = 1.83, p = 0.070), body weight (p = 0.146), and total cholesterol (t(98.55) = 1.90, p = 0.060) did not reach statistical significance. Average HRI did not differ significantly between groups (t(106.65) = 1.01, p = 0.316). Sex distribution (p = 0.126), hypertension status (p = 0.244), and current smoking status (p = 0.186) also did not differ significantly between groups.

HRI distribution and HRI-based steatosis grading

The mean average HRI in the complete cohort was 1.54 (SD 0.28), and the median was 1.51 (IQR, 1.28-1.78). Based on the protocol-defined HRI thresholds, 23 patients (19.8%) had mild steatosis, 52 (44.8%) had moderate steatosis, and 41 (35.3%) had severe steatosis. The distribution of HRI-defined steatosis grades did not differ significantly according to HbA1c-defined glycemic status (Pearson chi-square(2) = 3.47, p = 0.176; Table 2).

**Table 2:** Distribution of HRI-based steatosis severity by HbA1c-defined glycemic status. Association between HRI grade and HbA1c-defined glycemic status: Pearson chi-square(2) = 3.47, p = 0.176. HRI, hepatorenal index; SD, standard deviation

HRI grade	HRI threshold	Overall n (%)	Normal HbA1c n (%)	Dysglycemia n (%)	Average HRI, mean (SD)
Mild	1.05-1.24	23 (19.8)	10 (21.3)	13 (18.8)	1.164 (0.057)
Moderate	1.25-1.64	52 (44.8)	25 (53.2)	27 (39.1)	1.445 (0.110)
Severe	≥1.65	41 (35.3)	12 (25.5)	29 (42.0)	1.862 (0.114)

Comparison of HRI between normal HbA1c and dysglycemia groups

The median HRI was 1.48 (IQR 1.32-1.65) among patients with normal HbA1c and 1.52 (IQR 1.29-1.83) among patients with HbA1c-defined dysglycemia. A nonparametric sensitivity analysis showed no statistically significant difference in the HRI distributions (Mann-Whitney U = 1469, p = 0.393). This finding was consistent with the Welch t-test comparison in Table 1, which also showed no significant difference in mean HRI between groups (t(106.65) = 1.01, p = 0.316) (Figure 3).

**Figure 3:**
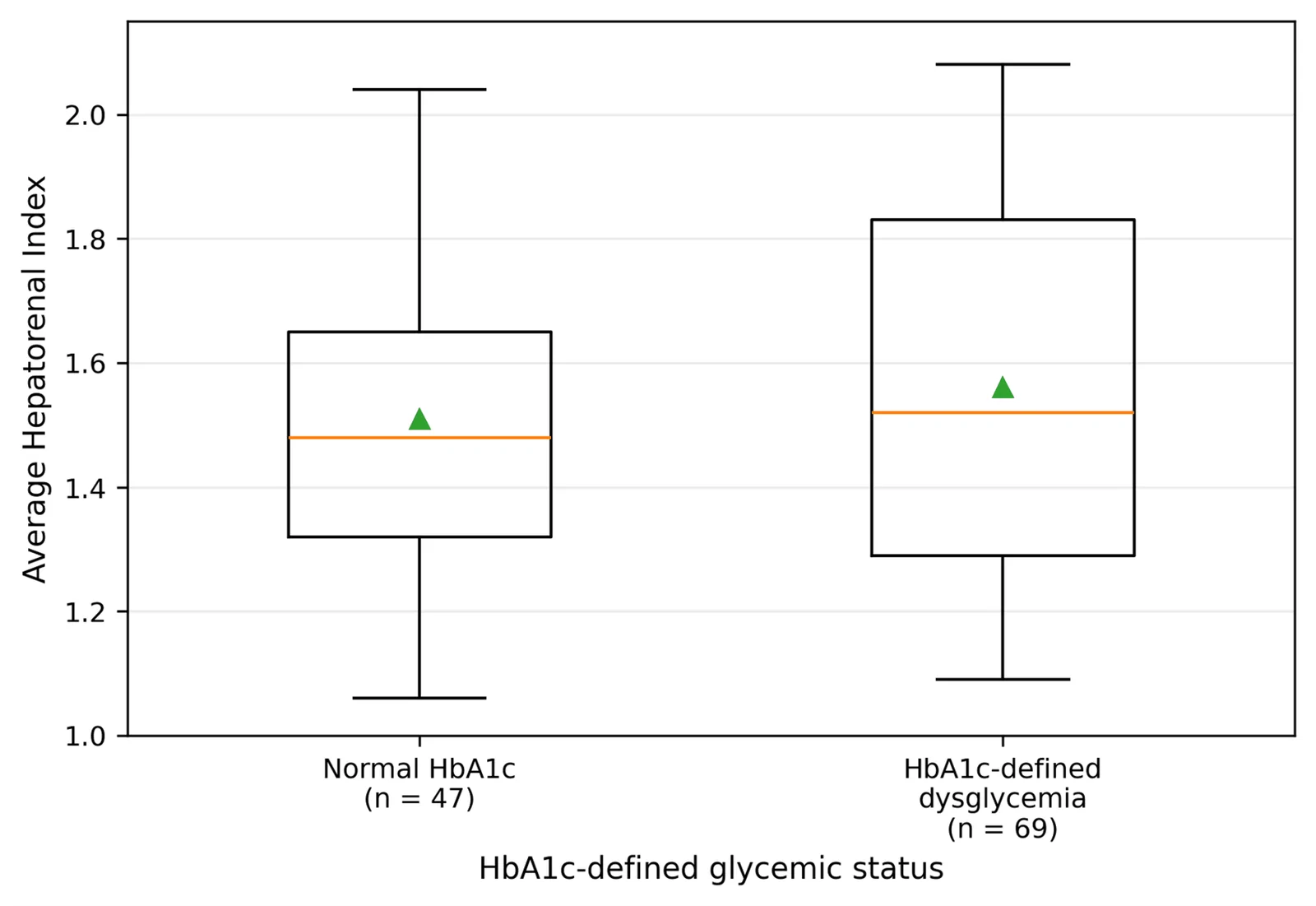
Distribution of average hepatorenal index by HbA1c-defined glycemic status. Boxplots show the median, interquartile range, whiskers, and mean marker.

Correlation of HRI with HbA1c and biochemical markers

Average HRI showed a weak positive Pearson correlation with HbA1c (r = 0.232, t(114) = 2.54, p = 0.012), whereas the corresponding Spearman rank correlation was not statistically significant (ρ = 0.111, p = 0.237). Average HRI was not significantly correlated with ALT, AST, total cholesterol, triglycerides, BMI, subcutaneous tissue thickness, or age (Table 3 and Figure 4). The discordance between Pearson and Spearman estimates prompted additional regression diagnostics and robust sensitivity analyses.

**Table 3:** Correlation of average HRI with HbA1c and biochemical/metabolic variables. HRI, hepatorenal index; ALT, alanine aminotransferase; AST, aspartate aminotransferase; BMI, body mass index

Variable	Pearson r	Pearson t statistic	Pearson p-value	Spearman rho	Spearman p-value
HbA1c, %	0.232	2.54	0.012	0.111	0.237
ALT, U/L	-0.047	0.51	0.614	-0.056	0.552
AST, U/L	0.035	0.37	0.713	0.019	0.841
Total cholesterol, mg/dL	0.015	0.16	0.874	-0.017	0.857
Triglycerides, mg/dL	0.012	0.13	0.899	0.054	0.562
BMI, kg/m²	0.126	1.36	0.178	0.087	0.355
Subcutaneous tissue thickness, mm	0.091	0.97	0.332	0.083	0.377
Age, years	0.049	0.52	0.604	0.051	0.586

**Figure 4:**
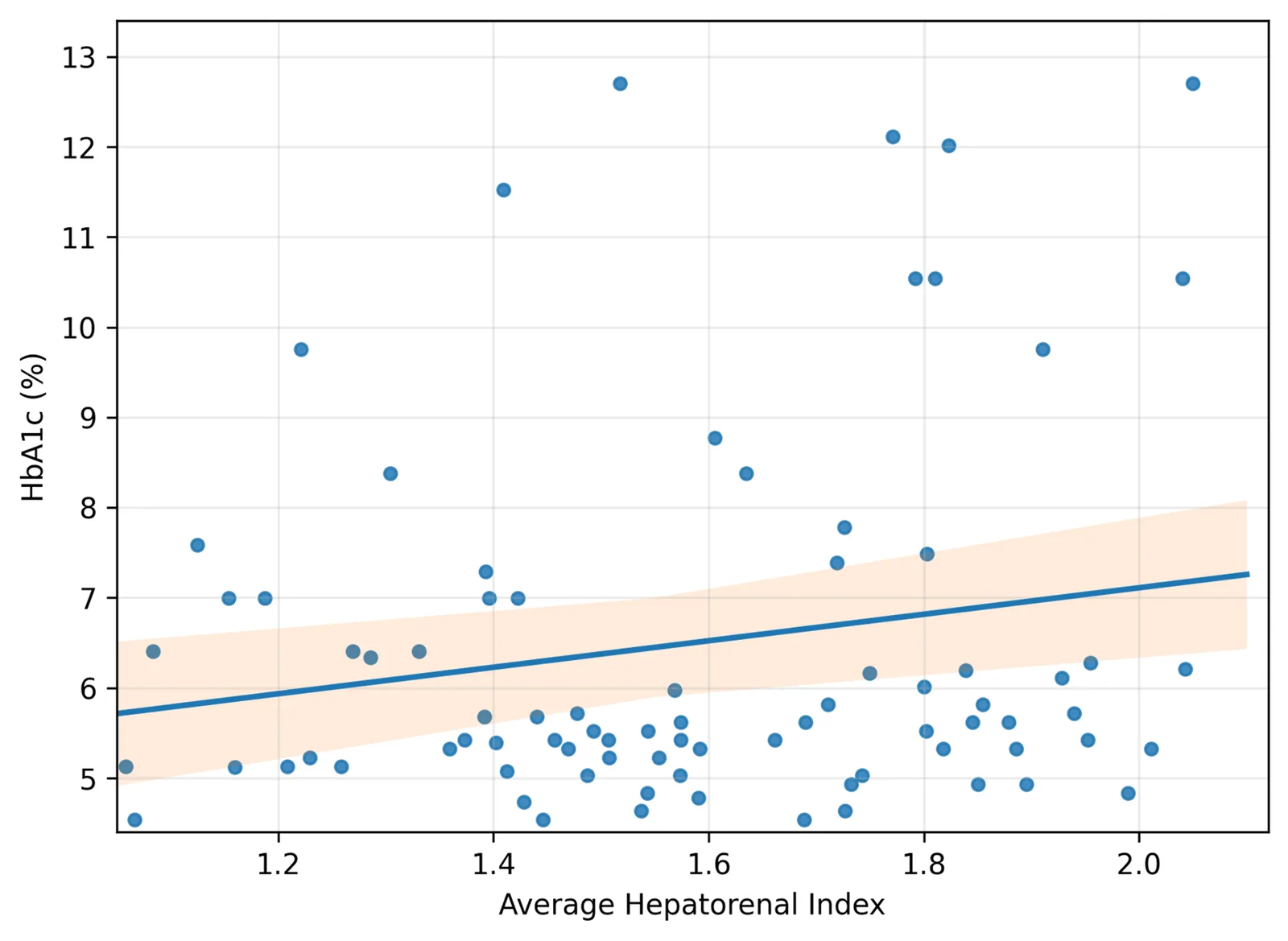
Scatter plot showing the relationship between average hepatorenal index and HbA1c. Pearson correlation showed a weak positive association (r = 0.232; p = 0.012), but the wide scatter indicates limited individual-level prediction.

Linear regression diagnostics and sensitivity analyses for HbA1c

In unadjusted OLS regression, average HRI was positively associated with HbA1c (β = 1.47, 95% CI 0.33 to 2.62; p = 0.012), but the model explained only 5.4% of HbA1c variance (R² = 0.054). After adjustment for age, sex, and BMI, the HRI coefficient remained statistically significant in the OLS model (β = 1.37, 95% CI 0.23 to 2.51; p = 0.019). The adjusted model explained 10.7% of HbA1c variance (R² = 0.107; adjusted R² = 0.074).

Regression diagnostics showed marked non-normality of residuals in both the unadjusted model (Shapiro-Wilk W = 0.783, p < 0.001) and the adjusted model (W = 0.784, p < 0.001). The unadjusted model showed evidence of heteroscedasticity (Breusch-Pagan chi-square = 6.69, p = 0.010), whereas this was not evident after adjustment (chi-square = 7.17, p = 0.127). Ten observations exceeded the conventional Cook's distance threshold of 4/n in the unadjusted model, indicating the presence of influential observations, several of which had high HbA1c values. There was no statistically significant evidence of a quadratic HRI-HbA1c relationship in either the unadjusted model (HRI-squared p = 0.219) or the adjusted model (HRI-squared p = 0.140).

Sensitivity analyses yielded method-dependent estimates. Using heteroscedasticity-consistent HC3 standard errors, the HRI coefficient remained statistically significant in both the unadjusted model (β = 1.47, 95% CI 0.20 to 2.75; p = 0.024) and the age-, sex-, and BMI-adjusted model (β = 1.37, 95% CI 0.10 to 2.65; p = 0.035). In contrast, Huber M-estimation, which downweights observations with large residual influence, attenuated the association and yielded nonsignificant HRI coefficients in the unadjusted model (β = 0.44, 95% CI -0.17 to 1.06; p = 0.160) and the adjusted model (β = 0.41, 95% CI -0.19 to 1.01; p = 0.180) (Table 4). Together with the nonsignificant Spearman correlation, these analyses indicate that the weak OLS/Pearson association was not robust across analytic approaches and should be interpreted cautiously.

**Table 4:** Linear regression analysis of HbA1c as a continuous outcome with robust sensitivity analyses. The adjusted models included age, sex, and BMI. HC3 denotes heteroscedasticity-consistent standard errors. Huber M-estimation was used as a robust regression sensitivity analysis. BMI, body mass index; CI, confidence interval; HRI, hepatorenal index; SE, standard error; OLS, ordinary least-squares

Analysis	Predictor	Beta coefficient	SE	95% CI	p-value	Model R²
Primary OLS, unadjusted	Average HRI	1.47	0.58	0.33 to 2.62	0.012	0.054
Primary OLS, adjusted	Average HRI	1.37	0.58	0.23 to 2.51	0.019	0.107
Primary OLS, adjusted	Age, per year	0.03	0.01	0.00 to 0.06	0.037	-
Primary OLS, adjusted	BMI, per kg/m²	0.01	0.04	-0.07 to 0.10	0.740	-
Primary OLS, adjusted	Male sex	-0.39	0.32	-1.03 to 0.25	0.233	-
HC3 sensitivity, unadjusted	Average HRI	1.47	0.64	0.20 to 2.75	0.024	0.054
HC3 sensitivity, adjusted	Average HRI	1.37	0.64	0.10 to 2.65	0.035	0.107
Huber robust, unadjusted	Average HRI	0.44	0.31	-0.17 to 1.06	0.160	Not applicable
Huber robust, adjusted	Average HRI	0.41	0.30	-0.19 to 1.01	0.180	Not applicable

Logistic regression analysis for HbA1c-defined dysglycemia

When HbA1c-defined dysglycemia was analyzed as a binary outcome, average HRI was not significantly associated with dysglycemia in the unadjusted model. The OR per 0.1-unit increase in HRI was 1.07 (95% CI 0.94 to 1.22; Wald chi-square(1) = 0.97, p = 0.325). After adjustment for age, sex, and BMI, HRI remained nonsignificant (adjusted OR per 0.1-unit increase, 1.05; 95% CI 0.91 to 1.21; Wald chi-square(1) = 0.45, p = 0.501). Age, BMI, and male sex also did not reach statistical significance individually in the adjusted model (Table 5). The full multivariable model had an overall likelihood-ratio chi-square(4) = 11.28 (p = 0.024).

**Table 5:** Logistic regression analysis for predictors of HbA1c-defined dysglycemia. The outcome was HbA1c-defined dysglycemia, defined as HbA1c ≥5.7%. The primary adjusted model included average HRI, age, sex, and BMI. Female sex was the reference category. BMI, body mass index; CI, confidence interval; HRI, hepatorenal index

Model	Predictor	Odds ratio	95% CI	Wald statistic (χ^2^)	p-value
Unadjusted	Average HRI, per 0.1-unit increase	1.07	0.94 to 1.22	0.97	0.325
Adjusted	Average HRI, per 0.1-unit increase	1.05	0.91 to 1.21	0.45	0.501
Adjusted	Age, per year	1.04	1.00 to 1.07	3.47	0.062
Adjusted	BMI, per kg/m²	1.11	0.99 to 1.25	3.35	0.067
Adjusted	Male sex	0.52	0.24 to 1.15	2.62	0.106

As an exploratory sensitivity analysis addressing additional measured metabolic and clinical factors, triglycerides, hypertension, subcutaneous tissue thickness, current smoking, ALT, and AST were added to the age-, sex-, and BMI-adjusted model. HRI remained unrelated to HbA1c-defined dysglycemia (OR per 0.1-unit increase = 1.00, 95% CI 0.86 to 1.17; p = 0.958). Because this expanded model included a relatively large number of predictors for the sample size, it was treated as a sensitivity analysis rather than the primary explanatory model.

ROC analysis and exploratory sample-derived HRI cutoff

ROC analysis demonstrated poor standalone discrimination of average HRI for HbA1c-defined dysglycemia. The AUC was 0.547 (95% CI 0.433 to 0.653; p = 0.393), close to the no-discrimination value of 0.50. The sample-derived Youden threshold was HRI ≥1.71. At this threshold, sensitivity was 40.6%, specificity was 78.7%, positive predictive value was 73.7%, negative predictive value was 47.4%, and overall accuracy was 56.0%. The positive likelihood ratio was 1.91 (95% CI 1.03 to 3.54), and the negative likelihood ratio was 0.75 (95% CI 0.59 to 0.96), indicating limited rule-in and rule-out performance (Figure 5 and Table 6). Because the threshold was selected and evaluated in the same 116 participants, it is reported only as a hypothesis-generating, sample-derived cutoff and should not be interpreted as a validated clinical threshold.

**Figure 5:**
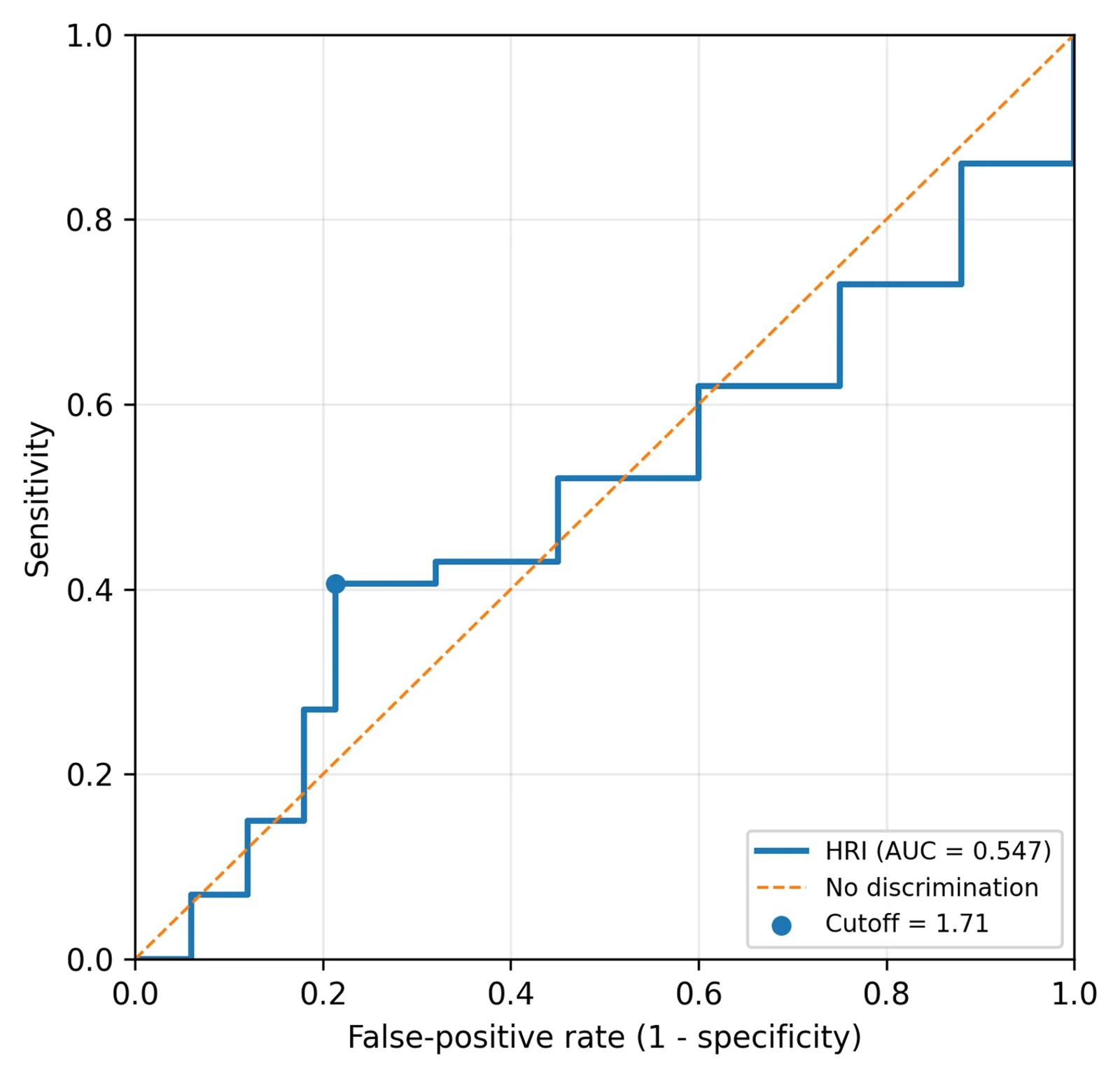
Receiver operating characteristic curve of average HRI for detecting HbA1c-defined dysglycemia. The AUC was 0.547 (95% CI 0.433 to 0.653), indicating poor standalone discriminative performance. HRI, hepatorenal index; AUC, area under the receiver operating characteristic curve

**Table 6:** ROC-derived diagnostic performance of average HRI for detecting HbA1c-defined dysglycemia. The HRI cutoff was derived and evaluated in the same cohort and is therefore exploratory. Confidence intervals for sensitivity, specificity, predictive values, and accuracy are Wilson intervals; likelihood-ratio confidence intervals are log-scale estimates. *Hypothesis-generating only AUC, area under the receiver operating characteristic curve; CI, confidence interval; HRI, hepatorenal index; ROC, receiver operating characteristic

Metric	Estimate	95% CI	Interpretation
AUC	0.547	0.433 to 0.653	Poor discrimination; p = 0.393
Optimal HRI cutoff	≥ 1.71	Not applicable	*Selected by Youden index
Sensitivity	40.6%	29.8% to 52.4%	Too low for standalone screening
Specificity	78.7%	65.1% to 88.0%	Moderate rule-in tendency
Positive predictive value	73.7%	58.0% to 85.0%	Influenced by high dysglycemia prevalence
Negative predictive value	47.4%	36.7% to 58.4%	Poor rule-out value
Accuracy	56.0%	47.0% to 64.7%	Limited overall classification
Positive likelihood ratio	1.91	1.03 to 3.54	Limited rule-in value
Negative likelihood ratio	0.75	0.59 to 0.96	Limited rule-out value

## Discussion

This cross-sectional study evaluated the relationship between ultrasound-derived HRI and HbA1c-defined dysglycemia among adults with ultrasonographically diagnosed NAFLD. The principal finding was that HRI showed, at most, a weak continuous association with HbA1c, and this association was not robust across different analytical approaches. More importantly, HRI demonstrated poor standalone discrimination for HbA1c-defined dysglycemia and was not independently associated with dysglycemia in logistic regression. These findings suggest that although hepatic steatosis and abnormalities in glucose metabolism are biologically related, the degree of liver echogenicity captured by HRI does not provide a reliable surrogate for biochemical glycemic assessment [14].

The distinction between statistical association and clinical discrimination is particularly important in interpreting the present findings. In conventional analysis, average HRI showed a weak positive Pearson correlation with HbA1c (r = 0.232, p = 0.012), and OLS regression demonstrated a statistically significant HRI coefficient in both the unadjusted and age-, sex-, and BMI-adjusted models. However, HRI explained only 5.4% of the variance in HbA1c in the unadjusted model and 10.7% in the adjusted model. The corresponding Spearman rank correlation was not statistically significant (rho = 0.111, p = 0.237). Thus, the statistically significant Pearson association should not be interpreted as evidence of a strong or clinically predictive relationship.

Additional regression diagnostics provided further explanation for this discordance. Residuals from the ordinary linear models showed marked departure from normality, and the unadjusted model demonstrated evidence of heteroscedasticity. Several observations, particularly participants with relatively high HbA1c values, were identified as influential using Cook's distance. There was no significant evidence of a quadratic HRI-HbA1c relationship, suggesting that the discrepancy was not explained simply by failure to model a curvilinear association. When heteroscedasticity-consistent HC3 standard errors were applied, the HRI coefficient remained statistically significant; however, Huber M-estimation, which reduces the influence of observations with large residuals, attenuated the association and produced nonsignificant HRI coefficients in both the unadjusted and adjusted models. Taken together with the nonsignificant Spearman correlation, these findings indicate that the weak Pearson/OLS association is method-sensitive and partly influenced by the distribution of HbA1c values. The appropriate interpretation is therefore that a weak continuous relationship may exist, but its magnitude and statistical significance are not sufficiently robust to support individual-level prediction.

This finding is biologically plausible. HRI represents the relative echogenicity of hepatic parenchyma compared with the renal cortex and is principally intended to quantify sonographic changes associated with hepatic fat accumulation. HbA1c, in contrast, reflects average systemic glycemic exposure over time and is determined by several processes beyond hepatic steatosis, including pancreatic beta-cell function, peripheral insulin sensitivity, adiposity, dietary exposure, physical activity, red-cell turnover, anemia, renal function, and other metabolic and hematological factors. Consequently, increasing hepatic fat-related echogenicity need not parallel increasing HbA1c in a linear or clinically discriminative manner. A quantitative imaging marker of steatosis and a biochemical marker of glycemia therefore measure related but fundamentally different aspects of metabolic disease.

The findings can also be placed in the context of previous HRI studies. Avramovski et al. evaluated 89 patients with NAFLD and reported impaired glucose metabolism in 48.31% of participants, with a positive association between HRI and impaired glucose metabolism. However, the proportion of HbA1c variation explained by HRI was modest, with an R² of approximately 0.058 for the HRI-HbA1c relationship [9]. This is relevant to the present results because it suggests that statistically detectable relationships between HRI and glycemic measures do not necessarily imply strong explanatory or diagnostic performance. Differences in study populations, definitions of impaired glucose metabolism, background disease severity, and analytical approaches may account for the stronger predictive interpretation reported in that earlier study. Isaksen et al. examined a related but distinct metabolic outcome by evaluating HRI as a predictor of insulin resistance measured using the homeostasis model assessment of insulin resistance (HOMA1-IR). In their cohort of 90 participants, an HRI threshold of approximately 1.17 showed high sensitivity for HOMA1-IR-defined insulin resistance, particularly among individuals with obesity [15,16]. These findings support an association between hepatic steatosis and insulin resistance but should not be considered directly equivalent to prediction of HbA1c-defined dysglycemia. Insulin resistance can precede measurable deterioration in HbA1c and may coexist with normoglycemia while compensatory pancreatic beta-cell function remains sufficient. Therefore, HRI may relate more closely to an insulin-resistant metabolic phenotype than to a binary HbA1c threshold.

More recent evidence also reinforces the distinction between the value of HRI for identifying hepatic steatosis and its potential use as a marker of systemic metabolic status. Hamadouche et al. prospectively evaluated HRI in 392 patients with type 2 diabetes and metabolic dysfunction-associated steatotic liver disease using magnetic resonance imaging proton density fat fraction (MRI-PDFF) as the reference standard [16]. HRI showed reasonably good performance for detecting the presence of steatosis, with an AUC of approximately 0.81 for detecting at least mild steatosis, but its performance declined substantially for discriminating higher grades of steatosis, with an AUC of approximately 0.61 for detecting at least moderate steatosis. These findings support the continued role of HRI as an accessible quantitative ultrasound marker of hepatic fat while also demonstrating limitations in its ability to distinguish increasingly complex disease phenotypes. The poor AUC observed for HbA1c-defined dysglycemia in the present study is therefore not inconsistent with HRI being useful for its intended imaging purpose.

Another important observation in the present cohort was that substantial sonographic steatosis was common even among participants with normal HbA1c. The normal-HbA1c group had a mean HRI of 1.51, and 37 of 47 participants (78.7%) were classified as having moderate or severe HRI-defined steatosis. Conversely, not all participants with dysglycemia demonstrated markedly elevated HRI. This overlap provides a direct explanation for the poor separation between glycemic groups. It also illustrates that normal HbA1c does not exclude substantial hepatic steatosis and, conversely, substantial steatosis does not necessarily indicate established dysglycemia. Because of the cross-sectional study design, however, it cannot be determined whether individuals with elevated HRI but normal HbA1c are at increased risk of subsequently developing prediabetes or diabetes. Prospective longitudinal studies would be required to evaluate this possibility.

The multivariable analyses further support the limited independent relationship between HRI and dysglycemia. After adjustment for age, sex, and BMI, HRI was not independently associated with HbA1c-defined dysglycemia. These variables were selected because age, sex, and adiposity can influence both hepatic steatosis and glucose regulation and therefore represent clinically plausible common determinants of the relationship under investigation. An expanded sensitivity model additionally incorporating triglycerides, hypertension, subcutaneous tissue thickness, smoking, ALT, and AST did not materially change the HRI association. This provides some reassurance that the negative relationship was not simply the consequence of omission of these measured factors. Nevertheless, residual confounding cannot be excluded because other relevant determinants of glycemic status, including dietary exposure, physical activity, visceral adiposity, insulin levels, and family history of diabetes, were not available. The expanded model must also be interpreted cautiously because the number of predictors was relatively large for the study sample, and some characteristics, particularly current smoking, were uncommon.

The ROC findings are clinically important. In our study, average HRI demonstrated an AUC of only 0.547 for identifying HbA1c-defined dysglycemia, indicating discrimination close to chance. The sample-derived Youden threshold of HRI ≥1.71 had a sensitivity of only 40.6%, despite a specificity of 78.7%. The corresponding positive likelihood ratio of 1.91 and negative likelihood ratio of 0.75 provide little clinically useful change in pre-test probability. Consequently, a substantial proportion of patients with HbA1c-defined dysglycemia would be missed if HRI were used as a screening test. Furthermore, because the 1.71 threshold was derived and evaluated in the same 116 participants, its apparent performance is susceptible to optimism. The cutoff should therefore be regarded solely as hypothesis-generating and not as a clinically validated threshold. Independent external validation would be required before any HRI threshold could be considered for metabolic risk stratification.

The absence of significant correlations between HRI and ALT, AST, total cholesterol, or triglycerides also emphasizes that these measures reflect different components of the metabolic and hepatic phenotype. HRI primarily quantifies ultrasound echogenicity associated with hepatic fat, whereas aminotransferase concentrations reflect hepatocellular injury and circulating lipid concentrations represent systemic lipid metabolism. Conventional grayscale ultrasonography therefore cannot characterize the full biological spectrum of steatotic liver disease. More advanced techniques, including MRI-PDFF and controlled attenuation parameter, provide more direct quantitative assessment of liver fat but are constrained by cost, equipment availability, expertise, and workflow requirements in many routine clinical settings [13,14]. HRI consequently retains practical value as an accessible quantitative adjunct to ultrasound, but its use should remain aligned with assessment of hepatic steatosis rather than extension to unrelated metabolic endpoints.

The broader metabolic context is also evolving with increasing recognition that obesity, dysglycemia, steatotic liver disease, and behavioral exposures such as alcohol consumption share overlapping neuroendocrine and metabolic pathways. Glucagon-like peptide-1 receptor agonists (GLP-1 RAs), originally developed for diabetes and subsequently widely used for obesity, illustrate this interconnected biology. A recent systematic review and meta-analysis by Eshraghi et al. reported reductions in several alcohol-related outcomes among individuals receiving GLP-1 RAs, with evidence suggesting effects on appetite, reward pathways, weight reduction, and metabolic regulation [17]. This evidence is not directly comparable with the present HRI study and should not be interpreted as supporting HRI as a marker of therapeutic response. Rather, it highlights the broader principle that steatotic liver disease, obesity, glycemic abnormalities, and alcohol-related behaviors arise within interacting metabolic pathways that cannot be represented adequately by a single imaging measurement. Comprehensive assessment of patients with steatotic liver disease therefore requires integration of imaging findings with biochemical, anthropometric, behavioral, and clinical information.

From a clinical perspective, the negative diagnostic finding is useful rather than merely null. Quantitative sonographic evidence of steatosis should not lead clinicians to infer the presence or absence of dysglycemia. Patients with NAFLD or the contemporary metabolic dysfunction-associated steatotic liver disease phenotype remain at substantial cardiometabolic risk and should undergo direct glycemic assessment irrespective of HRI value. Likewise, a relatively low HRI should not be used to reassure against impaired glucose metabolism. The present findings therefore support maintaining the separation between imaging-based assessment of hepatic steatosis and biochemical screening for metabolic disease. 

There are limitations that should nevertheless be acknowledged. First, the cross-sectional design precludes temporal or causal inference and cannot determine whether hepatic steatosis precedes worsening glycemic status. Second, HbA1c was the only available measure used to classify dysglycemia. Fasting plasma glucose, oral glucose tolerance testing, fasting insulin, and HOMA-IR were not assessed. This limits comparison with studies in which insulin resistance or oral glucose tolerance was the metabolic endpoint and may have resulted in misclassification in individuals whose HbA1c did not fully reflect glucose homeostasis. HbA1c may also be affected by anemia, hemoglobinopathies, renal dysfunction, and altered red-cell turnover.

Third, participants with previously diagnosed diabetes or current antidiabetic medication use were excluded. Consequently, HbA1c values ≥6.5% in this cohort represent diabetes-range glycemia rather than independently confirmed diabetes. Fourth, HRI remains operator-, machine-, and acquisition-dependent despite attempts to standardize ultrasound settings and ROI placement. Measurements were performed by a single experienced radiologist, but formal intra-observer and inter-observer reproducibility statistics were not available. Fifth, HRI was not validated against MRI-PDFF, controlled attenuation parameter, or histological quantification of liver fat in this cohort. Therefore, the study evaluates the metabolic association of protocol-derived HRI rather than its accuracy against a reference standard for hepatic steatosis.

The sample size also limits the precision of multivariable and subgroup analyses. Although the cohort contained 47 participants with normal HbA1c, 36 with prediabetes-range HbA1c, and 33 with diabetes-range HbA1c, these subgroups were too small for adequately powered multinomial or ordinal modeling. Combining prediabetes-range and diabetes-range HbA1c into a single dysglycemia outcome improved analytical stability but may have obscured differences across increasing degrees of glycemic abnormality. In addition, the sample-derived ROC threshold was evaluated in the same cohort in which it was identified and was not externally validated. Future studies should therefore include larger, multicenter populations; direct measures of insulin resistance and glucose tolerance; independent validation cohorts; standardized HRI reproducibility assessment; and reference imaging such as MRI-PDFF. Longitudinal studies would also be valuable for determining whether HRI has prognostic value for future dysglycemia even if its cross-sectional diagnostic performance is poor.

Taken together, the findings indicate that HRI captures only a limited component of the metabolic variation reflected by HbA1c. The weak Pearson and OLS association was not reproduced consistently across rank-based and robust analytical approaches, while binary logistic regression and ROC analysis provided no evidence of clinically useful discrimination for HbA1c-defined dysglycemia. HRI should therefore be interpreted primarily as a quantitative ultrasonographic marker of hepatic steatosis rather than as a surrogate marker of glycemic status. Direct biochemical screening for dysglycemia remains necessary in patients with NAFLD regardless of HRI value.

## Conclusions

In this single-center, hospital-based cohort of adults with ultrasonographically diagnosed NAFLD, HRI showed a weak continuous association with HbA1c but poor standalone discrimination of HbA1c-defined dysglycemia. The findings do not exclude a modest association; however, they indicate that HRI should not be used as a substitute for direct biochemical assessment of glycemic status. The internally derived HRI cutoff of 1.71 is exploratory and requires validation in larger, independent populations before any clinical application.
